# Alteration of the gut microbiota associated with childhood obesity by 16S rRNA gene sequencing

**DOI:** 10.7717/peerj.8317

**Published:** 2020-01-14

**Authors:** Xiaowei Chen, Haixiang Sun, Fei Jiang, Yan Shen, Xin Li, Xueju Hu, Xiaobing Shen, Pingmin Wei

**Affiliations:** 1 Key Laboratory of Environmental Medicine Engineering, Ministry of Education, School of Public Health, Southeast University, Nanjing, China; 2 Department of Epidemiology and Health Statistics, School of Public Health, Southeast University, Nanjing, China

**Keywords:** Gut microbiota, 16S rRNA gene sequencing, Childhood obesity, Bacterial compositions, Alpha diversity, Beta Diversity

## Abstract

**Background:**

Obesity is a global epidemic in the industrialized and developing world, and many children suffer from obesity-related complications. Gut microbiota dysbiosis might have significant effect on the development of obesity. The microbiota continues to develop through childhood and thus childhood may be the prime time for microbiota interventions to realize health promotion or disease prevention. Therefore, it is crucial to understand the structure and function of pediatric gut microbiota.

**Methods:**

According to the inclusion criteria and exclusion criteria, twenty-three normal weight and twenty-eight obese children were recruited from Nanjing, China. Genomic DNA was extracted from fecal samples. The V4 region of the bacterial 16S rDNA was amplified by PCR, and sequencing was applied to analyze the gut microbiota diversity and composition using the Illumina HiSeq 2500 platform.

**Results:**

The number of operational taxonomic units (OTUs) showed a decrease in the diversity of gut microbiota with increasing body weight. The alpha diversity indices showed that the normal weight group had higher abundance and observed species than the obese group (Chao1: *P* < 0.001; observed species: *P* < 0.001; PD whole tree: *P* < 0.001; Shannon index: *P* = 0.008). Principal coordinate analysis (PCoA) and Nonmetric multidimensional scaling (NMDS) revealed significant differences in gut microbial community structure between the normal weight group and the obese group. The liner discriminant analysis (LDA) effect size (LEfSe) analysis showed that fifty-five species of bacteria were abundant in the fecal samples of the normal weight group and forty-five species of bacteria were abundant in the obese group. In regard to phyla, the gut microbiota in the obese group had lower proportions of Bacteroidetes (51.35%) compared to the normal weight group (55.48%) (*P* = 0.030). There was no statistical difference in Firmicutes between the two groups (*P* = 0.436), and the Firmicutes/Bacteroidetes between the two groups had no statistical difference (*P* = 0.983). At the genus level, *Faecalibacterium, Phascolarctobacterium, Lachnospira*, *Megamonas*, and *Haemophilus* were significantly more abundant in the obese group than in the normal weight group (*P* = 0.048, *P* = 0.018, *P* < 0.001, *P* = 0.040, and *P* = 0.003, respectively). The fecal microbiota of children in the obese group had lower proportions of *Oscillospira* and *Dialister* compared to the normal weight group (*P* = 0.002 and *P* = 0.002, respectively).

**Conclusions:**

Our results showed a decrease in gut microbiota abundance and diversity as the BMI increased. Variations in the bacterial community structure were associated with obesity. Gut microbiota dysbiosis might play a crucial part in the development of obesity in Chinese children.

## Introduction

Obesity has become a global epidemic in the industrialized and developing world (Luke et al., 2014; Popkin, Adair & Ng, 2012). The World Health Organization’s briefing on obesity showed that global obesity is on a rapid upward trend and has doubled since 1980, with more than 40 million obese children in the word (Women, 2011). Obesity is associated with serious health risks, raising great concerns about multiple comorbidities occurring with obesity, including musculoskeletal disorders, type 2 diabetes, cardiovascular diseases, nonalcoholic fatty liver disease, and certain cancers (Dietrich & Hellerbrand, 2014; Farni et al., 2014; Global Burden of Metabolic Risk Factors for Chronic Disease Collaboration, 2014; Pettitt et al., 2014; Prospective Studies Collaboration, 2009; Wormser et al., 2011; Saydah et al., 2014). Due to the health and economic burden brought by the rising BMI, obesity has been included as a global noncommunicable diseases (NCD) target, halting the rise of obesity in 2025 to its 2010 level (Kontis et al., 2014).

Obesity prevails among children and results in a global problem regarding children’s general health and well-being. With high prevalence of childhood obesity, many children suffer from obesity-related complications (Han, Lawlor & Kimm, 2010). The pathogenesis of obesity is complicated, with multiple factors involved. Apart from genetic and nutritional factors, a new factor has been recently identified related to the onset and progression of obesity—the gut microbiota (Ridaura et al., 2013; Sonnenburg & Sonnenburg, 2014). As 500-1,000 species of microbes lives in the gastrointestinal tract, the gut microbiota is an ecosystem in itself (Karlsson et al., 2013a; Karlsson, Nookaew & Nielsen, 2014). The number of microbial genes in the gut microbiota is at least 150-fold larger than that of the human genes inside in human body (Qin et al., 2010).

Up to now, many studies have demonstrated that the gut microbiota, as the largest and most complex microecosystem inside the human body, plays extremely important roles in health and disease (Guyton & Alverdy, 2017; Leung et al., 2016; Lathrop et al., 2011; Nicholson et al., 2012; Sandhu et al., 2017; Tang & Hazen, 2016; Tremaroli & Backhed, 2012). The gut microbiota genome maintains normal physiological and metabolic functions in the human body, aiding food digestion through significantly enriching genes in metabolizing carbohydrates, vitamins, short-chain fatty acids and amino acids (Gill et al., 2006). For a long period, the microorganisms of the gut microbiota stay in the mutualistic symbioses and create a balanced and stable intestinal microsystem. This balanced microsystem prepare the host to adapt to special conditions, while the dysbiosis of this microsystem shall cause the compositional and functional imbalance in the intestinal microorganisms (Lynch & Pedersen, 2016).

In recent years, a growing number of researchers have paid attention to the gut microbiota and explored its functions in the pathogenesis and regulation of metabolic disorders. It has been suggested that the microbiota continues to develop through childhood and children may be the best candidate for microbiota interventions to realize health promotion or disease prevention (Hollister et al., 2015). Therefore, it is essential to foster a preliminary understanding of the pediatric gut microbiota. However, due to limited information of the pediatric gut microbiota regarding its structure and function, the mechanism and the degree of gut microbiota contributing to the development of childhood obesity have not yet been elucidated. As a result, a gut microbiota study in the context of obesity is needed to explain the relationship between the obesity epidemic and the gut microbiota.

Though it is difficult to cultivate the anaerobic gut microbiota in the laboratory, new genome sequencing techniques enable us to collect and analyze information of human gut microbiota from the perspectives of microbial composition and function (MetaHIT Consortium, 2011; Qin et al., 2010). In our study, we recruited children from a same geographic area to minimize variations irrelevant to obesity. Our research goal was to evaluate gut microbial biodiversity between obese and normal weight children, aged between 6-11 years old, using 16S rRNA gene sequencing. We expect that our findings could have some reference meanings in preventing and treating childhood obesity.

## Materials and Methods

### Ethics statement

The study was conducted in accordance to the *Declaration of Helsinki* revised in 2013 and approved by the ethics committee of Zhongda Hospital, Southeast University (approval number: 2017ZDSYLL109-P01). Participation in this study was voluntary, and all parents gave written informed consent.

### Research object

A total of 51 volunteers of both sexes (27 males and 24 females) were recruited from Nanjing, China. Subjects were strictly categorized into the normal weight group (*n* = 23) or the obese group (*n* = 28) according to the inclusion and exclusion criteria.

Inclusion criteria: (1) The age range for participation was 6-11 years old. (2) In accord with the determination standard of normal weight and obesity of children, the standard was “*Body mass index cut-offs for overweight and obesity in Chinese children and adolescents aged 2-18 years*” (Li et al., 2010) formulated by the Department of Growth and Development, Capital Institution of Pediatrics (Table 1). (3) Willing to participate in the study and obtain the consent of the guardian, voluntarily taking the subject and signing the informed consent form.

**Table 1:** Body mass index cut-offs for overweight and obesity in Chinese children and adolescents aged 2-18 years.

Age (Years)	Boys		Girls	
	Overweight	Obesity	Overweight	Obesity
2	17.5	18.9	17.5	18.9
3	16.8	18.1	16.9	18.3
4	16.5	17.8	16.7	18.1
5	16.5	17.9	16.6	18.2
6	16.8	18.4	16.7	18.4
7	17.2	19.2	16.9	18.8
8	17.8	20.1	17.3	19.5
9	18.5	21.1	17.9	20.4
10	19.3	22.2	18.7	21.5
11	20.1	23.2	19.6	22.7
12	20.8	24.2	20.5	23.9
13	21.5	25.1	21.4	25.0
14	22.1	25.8	22.2	25.9
15	22.7	26.5	22.8	26.7
16	23.2	27.0	23.3	27.2
17	23.6	27.5	23.7	27.6
18	24.0	28.0	24.0	28.0

Exclusion criteria: (1) Antibiotics have been used in the past 4 weeks. (2) Gastrointestinal dysfunction, previous gastrointestinal disease history or diarrhea, abdominal distension, abdominal pain or constipation within the past 4 weeks. (3) Trauma, serious infection, and infectious diseases. (4) Hereditary obesity. (5) Drug-induced obesity. (6) Endocrine disorders and metabolic diseases.

### Sample collection

Fecal samples were collected and well-sealed in sterile boxes, immediately frozen in a refrigerator and transported to school the next morning and stored at −20 °C before being transferred in insulating polystyrene foam containers to the Key Laboratory of Environmental Medical Engineering and Education Ministry, where samples were stored at −80 °C until further analysis.

### DNA extraction and PCR amplification

Genomic DNA was extracted according to the specifications of TIANamp Stool DNA kit (TIANGEN, China, Cat. DP328), applied to all feces samples. The integrity and purity of DNA were detected by 1% agarose gel electrophoresis, while the concentration and purity of DNA were detected by NanoDrop One. The V4 region of the bacterial 16S rDNA was amplified by PCR with the specific primers 515F (5′-GTGCCAGCMGCCGCGGTAA-3′) and 806R (5′-GGACTACHVGGGTWTCTAAT-3′) labeled in a 12 bp barcode. PCR amplification was conducted using primers with a barcode and Premix Taq under the following conditions: 5 min at 94 °C for initialization, 30 s denaturation at 94 °C for 30 cycles, 30 s annealing at 52 °C, and 30 s extension at 72 °C, and a final 10 min elongation at 72 °C. The fragment length and concentration of PCR products were detected by 1% agarose gel electrophoresis, and samples with a main band length in the range of 290-310 bp were selected. After comparing the concentrations of PCR products using GeneTools Analysis Software (Version 4.03.05.0, SynGene), the required volume of each sample was calculated according to the principle of equal quality, and each PCR product was mixed and recovered by the An E.Z.N.A.^®^ Gel Extraction Kit (OMEGA, USA, Cat. D2500). TE buffer was used to elute and recover the target DNA fragments.

### Sequencing and data processing

Libraries were built according to the NEBNext^®^ Ultra™ DNA Library Prep Kit for Illumina^®^ and sequenced on an Illumina HiSeq2500 platform (Caporaso et al., 2011), and then 250 bp paired-end reads were generated. Trimmomatic software (V0.33, http://www.usadellab.org/cms/?page=trimmomatic) (Bolger, Lohse & Usadel, 2014) was used to filter the quality of the raw reads data at both ends. At the same time, with reference to the barcode and primer information at both ends of the sequence, Mothur software (V1.35.1, http://www.mothur.org) (Schloss et al., 2009) was used to distribute the sequence to corresponding samples; the allowed mismatch number of barcodes was 2, and the maximum mismatch number of primers was 3. Then, after quality control, barcode and primers were removed to obtain paired-end clean reads. For double-ended sequencing data, it was necessary to splice each pair of paired-end reads using FLASH software (V1.2.11, https://ccb.jhu.edu/software/FLASH/) (Magoc & Salzberg, 2011) in terms of the overlap between paired-end reads to splice the paired reads into a sequence and to filter out nonconforming tags in order to collect the original spliced sequence (raw tags). The minimum overlap length was set to 10 bp and the maximum allowable mismatch ratio in the overlap region of the spliced sequence was set to 0.1. Mothur software was used to carry out quality control and filter the spliced sequences to obtain effective spliced fragments.

### OTU and species community analysis

The USEARCH software (V8.0.1517, http://www.drive5.com/usearch/) (Edgar, 2010) was used to cluster all clean tags for all samples. By default, the sequence was clustered into an operational taxonomic unit (OTU) with 97% identity. Singleton OTUs were removed with usearch (http://www.drive5.com/usearch/manual/chimera_formation.html), and chimeric sequences were removed with UCHIME (http://www.drive5.com/usearch/manual/uchime_algo.html) (Edgar et al., 2011). The assign_taxonomy.py script and Ribosomal Database Project (RDP) Classifier method (Cole et al., 2007) in Quantitative Insights Into Microbial Ecology (QIIME, version 1.9.1) were used to obtain species annotation information. The number of valid tag sequences (No. of seqs) and the OTU taxonomic comprehensive information table (otu_table) were obtained by removing chloroplast and mitochondrial sequences as well as OTUs and the tags that could not be annotated at the set limit. Based on otu_table, R software (V2.15.3) (R Core Team, 2013) was used to calculate the annotation proportion of OTUs at each classification level, and the sequence of each sample at each classification level was obtained to form a column chart. All values greater than 0 in each column of values were recorded as 1 and summed, which was the total OTUs of each sample. On the basis of the normalized otu_table, the common or unique OTU analysis was conducted with the ggplot2 package in R software, and meanwhile the OTU triangulation was drawn using the ggtern package to show the common or unique OTUs and their abundance between the two groups. The pheatmap package in R software was used to carry out the cluster analysis between samples and species. With the phylogenetic relationship and relative abundance information of each OTU in the samples, the species annotation results of a single sample was visualized by KRONA software (http://sourceforge.net/projects/krona/) (Ondov, Bergman & Phillippy, 2011). Using GraPhlAn software (http://huttenhower.sph.harvard.edu/graphlan) (Asnicar et al., 2015), a single sample OTU annotation circle graph based on GraPhlAn was obtained. Representative OTU sequences with the top 50 overall relative abundance and genus classification information were selected, and Mafft software (Katoh et al., 2002) was used to carry out a multisequence comparison. FastTree software (http://microbesonline.org/fasttree/) (Price, Dehal & Arkin, 2009) was used to build trees simultaneously combining the relative abundance of each OTU and species annotation confidence information of representative sequences, and the ggtree software package (Yu et al., 2017) was used to carry out the visual display. OTU abundance information were normalized using a standard of sequence number corresponding to the sample with the least sequences. Based on the normalized data, alpha diversity and beta diversity were all performed in the following paragraphs.

### Alpha diversity analysis

According to the normalized OTU abundance table, the alpha_diversity.py script (http://huttenhower.sph.harvard.edu/graphlan) in the QIIME software package (version 1.9.1) (Caporaso et al., 2010; Kuczynski et al., 2011) was used to calculate four diversity indices (Chao et al., 2010). According to the OTU abundance table, the alpha_rarefaction.py script (http://qiime.org/scripts/alpha_rarefaction.html) in the QIIME software package was used to calculate dilution curve data of four diversity indices, and the vegan package (Dixon, 2003) was used to draw the dilution curve. Based on the OTU abundance table, the plot_rank_abundance_graph.py script (http://qiime.org/scripts/plot_rank_abundance_graph.html) in the QIIME software package was selected to calculate the rank debt curve index. The data from the specaccum species accumulation curve analysis was calculated and mapped in line with the normalized OTU abundance table.

### Beta diversity analysis

According to the normalized OTU abundance table, the beta diversity distance was calculated using jackknifed_beta_diversity.py script (http://qiime.org/scripts/jackknifed_beta_diversity.html) in QIIME software. Principal Coordinate Analysis (PCoA) was performed to get principal coordinates and visualize from complex data (Lozupone et al., 2011). A distance matrix of unweighted Unifrac among samples obtained before was transformed to a new set of orthogonal axes, the maximum variation factor is demonstrated by first principal coordinate, the second maximum one by the second principal coordinate, and so on. PCoA analysis was displayed by QIIME2 and ggplot2 package (Ginestet, 2011). Upgma_cluster.py script (http://qiime.org/scripts/upgma_cluster.html) in QIIME software was applied to build the cluster tree of samples using the UPGMA cluster analysis method. Based on the normalized OUT table (otu_table_subsampled), Nonmetric Multidimensional Scaling (NMDS) analysis was performed with the vegan package and displayed with the ggplot2 package in R software.

### LEfSe and PICRUSt analysis

The linear discriminant analysis (LDA) effect size (LEfSe) analyses were performed on the website http://huttenhower.sph.harvard.edu/galaxy (Segata et al., 2011). For OTUs with an average abundance in all samples that was greater than 0.1%, abundances were normalized to the sum of the values per sample in 1 million and then subjected to LDA. The cut off value was the absolute LDA score (log10) >2.0. Functional capacity of gut microbiota was predicted using the Phylogenetic Investigation of Communities by Reconstruction of Unobserved States (PICRUSt) (Galaxy Version 1.0.0) (Langille et al., 2013). The closed reference OTU table was generated from quality control reads in QIIME against the Greengenes reference sequence database. Closed OTU-table drawn by QIIME was compared with KEGG (Kyoto Encyclopedia of Genes and Genomes) database to obtain functional predictions. PICRUSt predictions were categorized as levels 1-3 into KEGG pathways.

### Statistical analysis

IBM SPSS 23.0 software were applied in all statistical analyses. Depending on the normality of the underlying data drawn from the Shapiro-Wilk test, differences between groups were examined by two-tailed *t*-test or Wilcoxon rank-sum test. Test results at an alpha of *P* < 0.05 were considered statistically significant.

## Results

### Study population

In this study, we categorized 28 obese children into the obese group and 23 healthy children into the normal weight group to analyze the two group’s gut microbial composition. The two groups revealed no statistical difference regarding gender ratio and age (Table 2).

**Table 2:** Characteristics of the study population.

Variables	Normal weight (*n* = 23) Mean (SD)	Obesity (*n* = 28) Mean (SD)
Gender (Boys/Girls)	11/12	16/12
Age (Years)	8.86(1.61)	8.49(1.48)
BMI (kg/m^2^)	15.32(1.42)	24.44(2.03)^*^

**Notes.**

* Asterisks indicate statistical significance (^∗^*P* < 0.05, Wilcoxon rank-sum test).

### Sequencing data

A total of 2,349,074 raw sequence reads were obtained from the 51 subjects. After a series of quality filtering, 2,004,646 classifiable 16S rRNA gene sequences were obtained, and the average number of sequences for each individual was 39,307 (ranging from 25,813 to 55,847). All sequences were clustered with representative sequences, and a 97% sequence identity cut-off was used.

**Figure 1:**
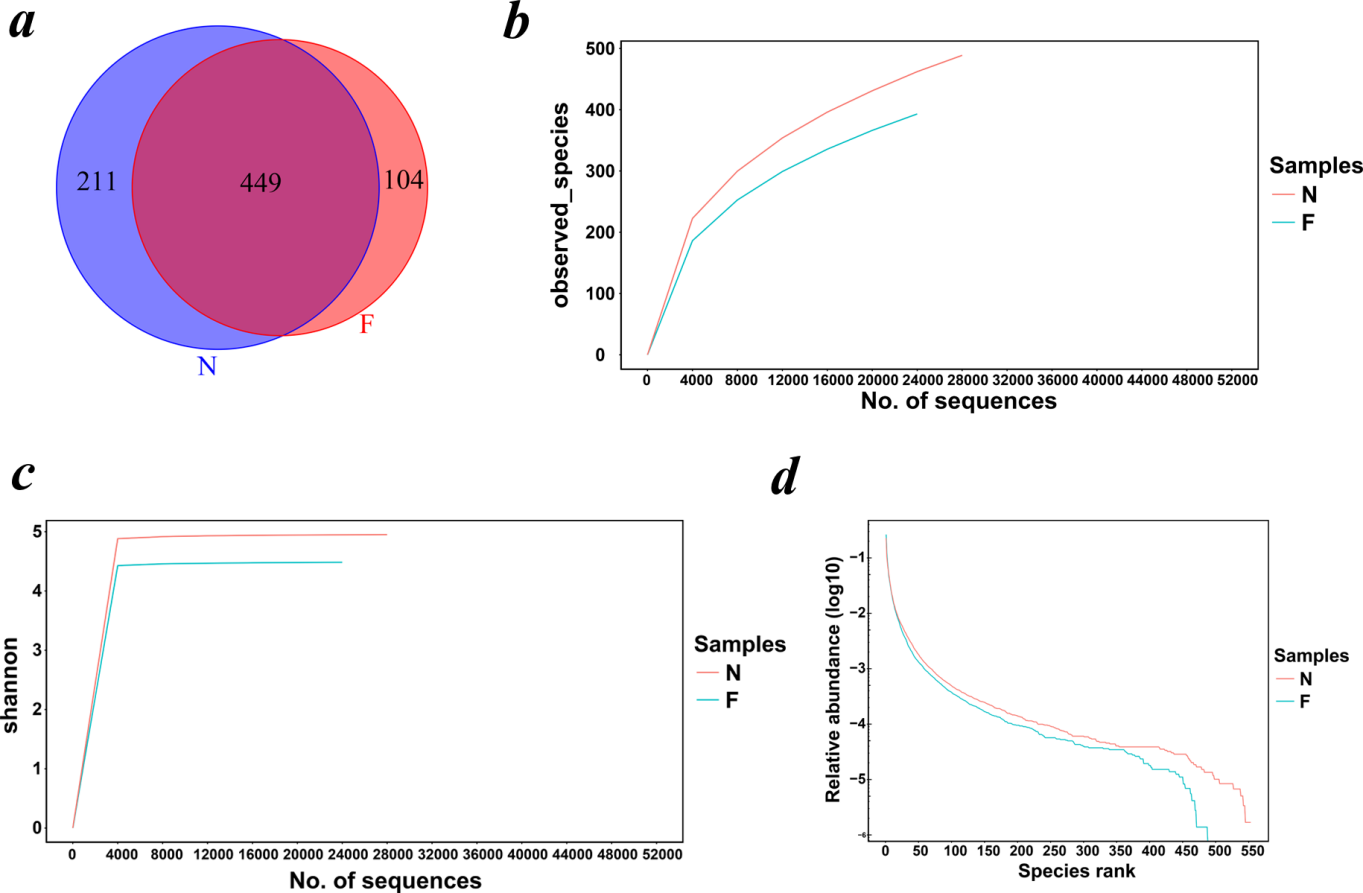
Venn diagram and rarefaction curves for calculated OTUs. (A) Different colors represent different groups in the Venn diagram. The areas with overlapping circles of different colors represent the set of OTUs commonly present in the counterpart groups, and the single-layer zone represents the number of OTUs uniquely found in each group. (B) Observed species curves and (C) Shannon curves representing the observed number of species in the two groups. The abscissa represents the number of sequences extracted by resampling, and the ordinate indicates the diversity value or the average number of OTUs per sample in each group. Rarefaction curves show the observed species at various sequencing depths. (D) The rank-abundance curve shows the species richness and uniformity. The curve width indicates that the species composition was more abundant. A flat curve indicates a more uniform species composition. (N: normal weight group; F: obese group).

The Venn diagram (Fig. 1A) demonstrated the shared and exclusive communities between the groups. The total number of OTUs obtained was 1,213, among which 449 OTUs were shared by both groups, 211 genera were specific to the normal weight group and 104 genera were specific to the obese group, showing a decrease in the gut microbial diversity as the body weight increased.

### Estimation of the alpha diversity and beta diversity

**Figure 2:**
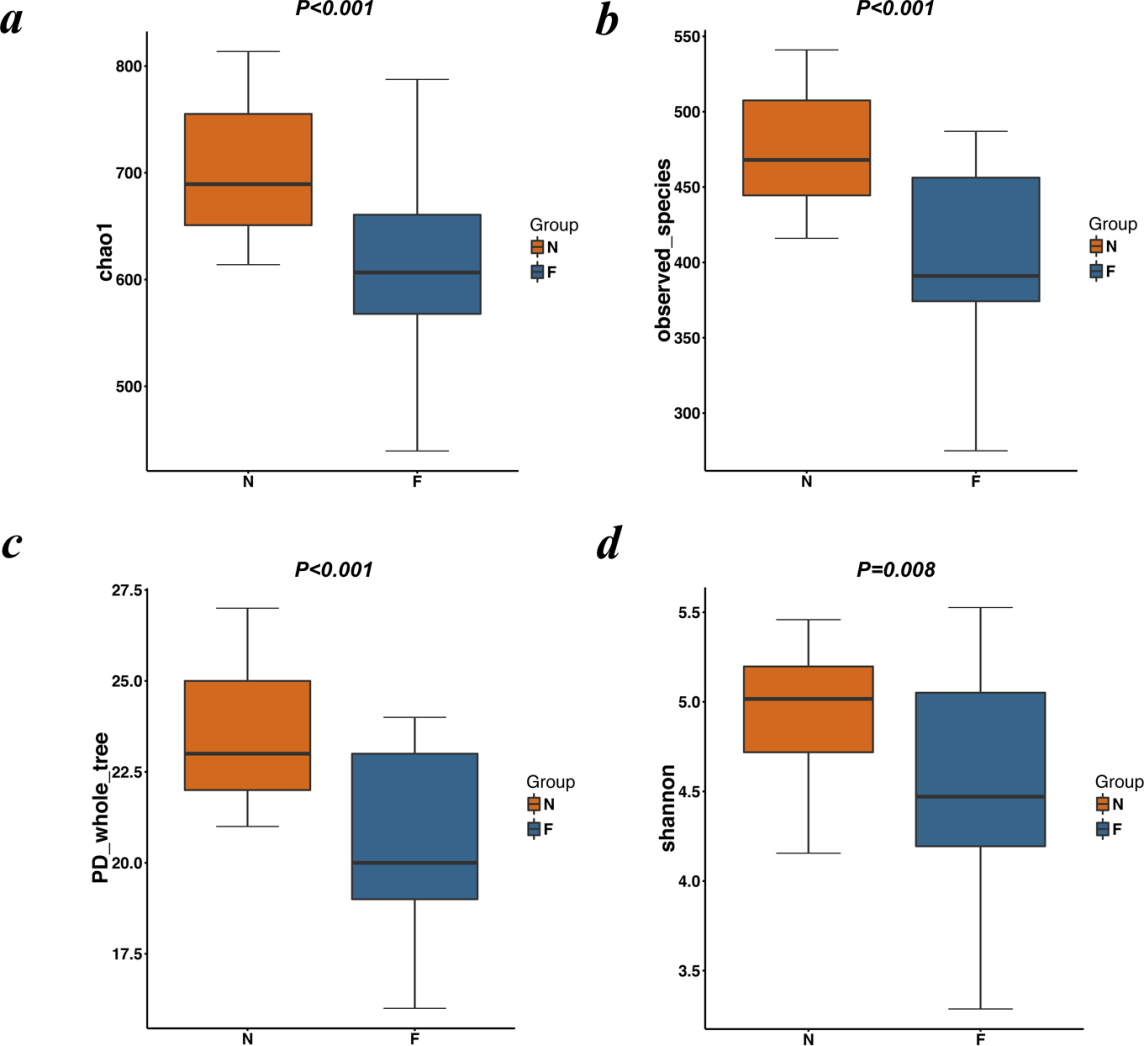
Alpha diversity metrics (Chao1 index, observed species, PD whole tree and Simpson index) of OTU-level fecal bacterial communities. (A) (B) Boxplotsfor comparison of species richness (Chao1 index; observed species) between the two study groups; (C) Boxplots for comparison of phylogenetic diversity (PD whole tree); (D) Boxplots for comparison of species diversity (Shannon index). All Boxplots show that the normal weight group had more abundance and diversity than the obese group. (N: normal weight group; F: obese group).

Rarefaction curves drawn on the observed species and Shannon indices (Figs. 1B and 1C) indicated that although deeper sequencing may reveal rare OTUs, the majority of microbial diversity had been captured. These curves also revealed that the alpha diversity in samples from the normal-weight group was the highest and that in obesity group was the lowest. To confirm its validity, we calculated Chao1 indices by Wilcoxon rank-sum test (Fig. 2), finding that the mean microbial abundance between two groups decreased significantly (*P* = 0.006, Wilcoxon rank-sum test). Shown in the rank-abundance curve, the species abundance and uniformity of gut microbiota in the obese group were lower than those in the normal weight group (Fig. 1D).

**Figure 3:**
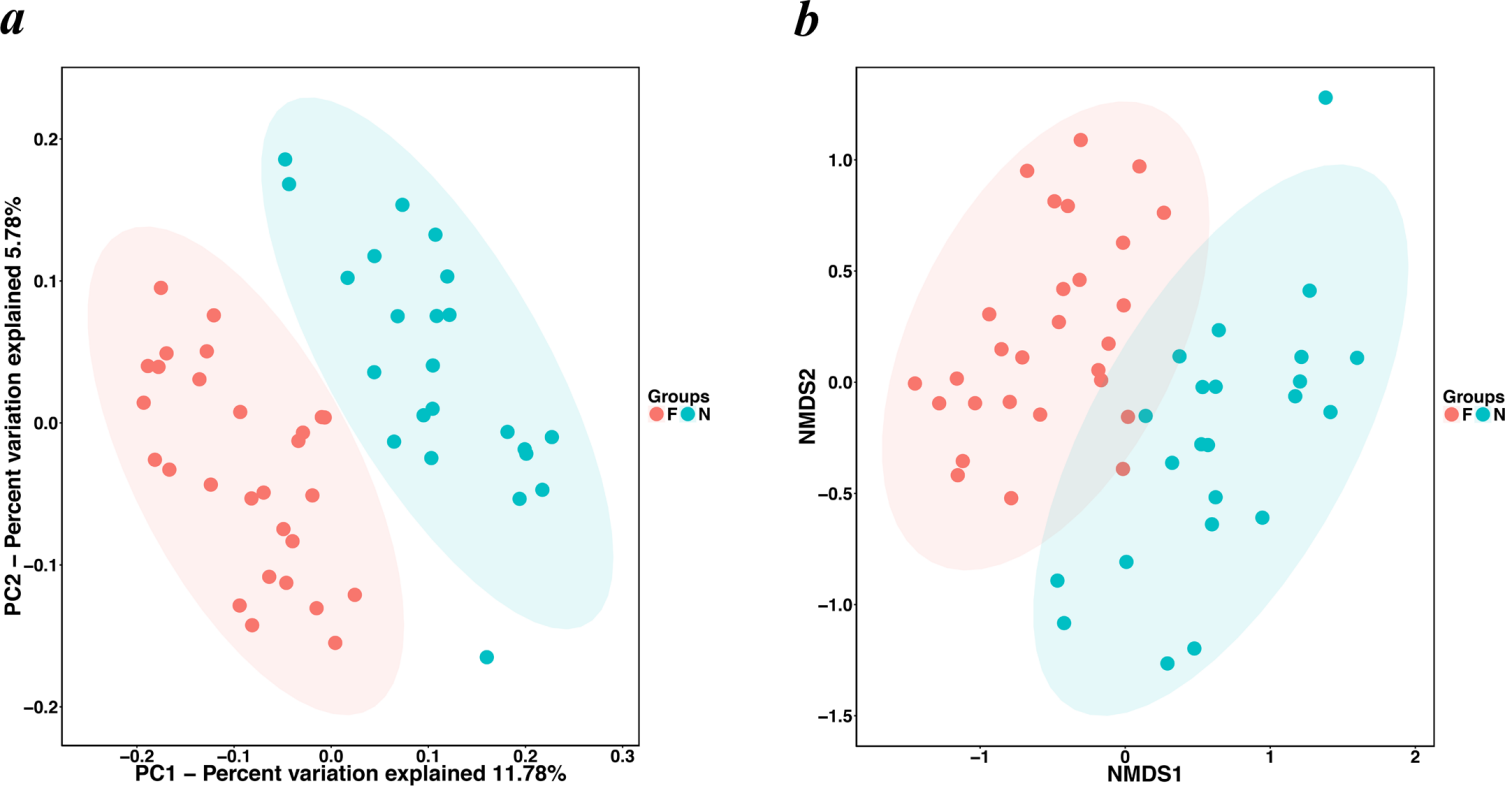
PCoA and NMDS based on the abundance of OTUs. (A) PCoA and (B) NMDS plots comparing sample distribution between the two groups. Points clustered at the left and the right left represent the gut microbial composition of the obese group and the normal weight group, respectively. The closer the spatial distance of the sample, the more similar the species composition of the sample. (N: normal weight group; F: obese group).

The alpha diversity indices (the Chao1, observed species, PD whole tree and Shannon index) were used to describe alpha diversity (Fig. 2). Significant differences were found between the normal weight group and the obese group (*P* < 0.001; *P* <  0.001; *P* < 0.001; and *P* = 0.008, respectively, Wilcoxon rank-sum test), showing a higher abundance and diversity in the normal weight group than in the obese group. In addition to the alpha diversity evaluation, the unweighted UniFrac analysis was applied to compare similarities among gut microbial communities (beta diversity). Looking into the bacterial composition profiles, we observed significant differences between the normal weight group and the obese group (*R* ^2^ = 0.054, *P* = 0.001, adonis analysis). NMDS and PCoA based on the abundance of OTUs revealed differences in the microbial composition (Fig. 3). Specifically, an evident clustering was identified for subjects of normal weight and obesity. The observation revealed significant differences in gut microbial community structure between the normal weight group and the obese group, with two principal component scores accounted respectively for 11.59% and 5.57% of the total variations. Moreover, separation between the two groups was particularly obvious. Representing the intestinal microbial composition, data points for subjects of normal weight clustered at the right and obese ones at the left.

### The relative abundance of fecal bacterial communities

Statistics of the OTUs suggested the relative abundance of the bacteria at the categorization of phylum, class, order, family and genus. The results showed that fecal bacterial composition differed between the two groups.

**Figure 4:**
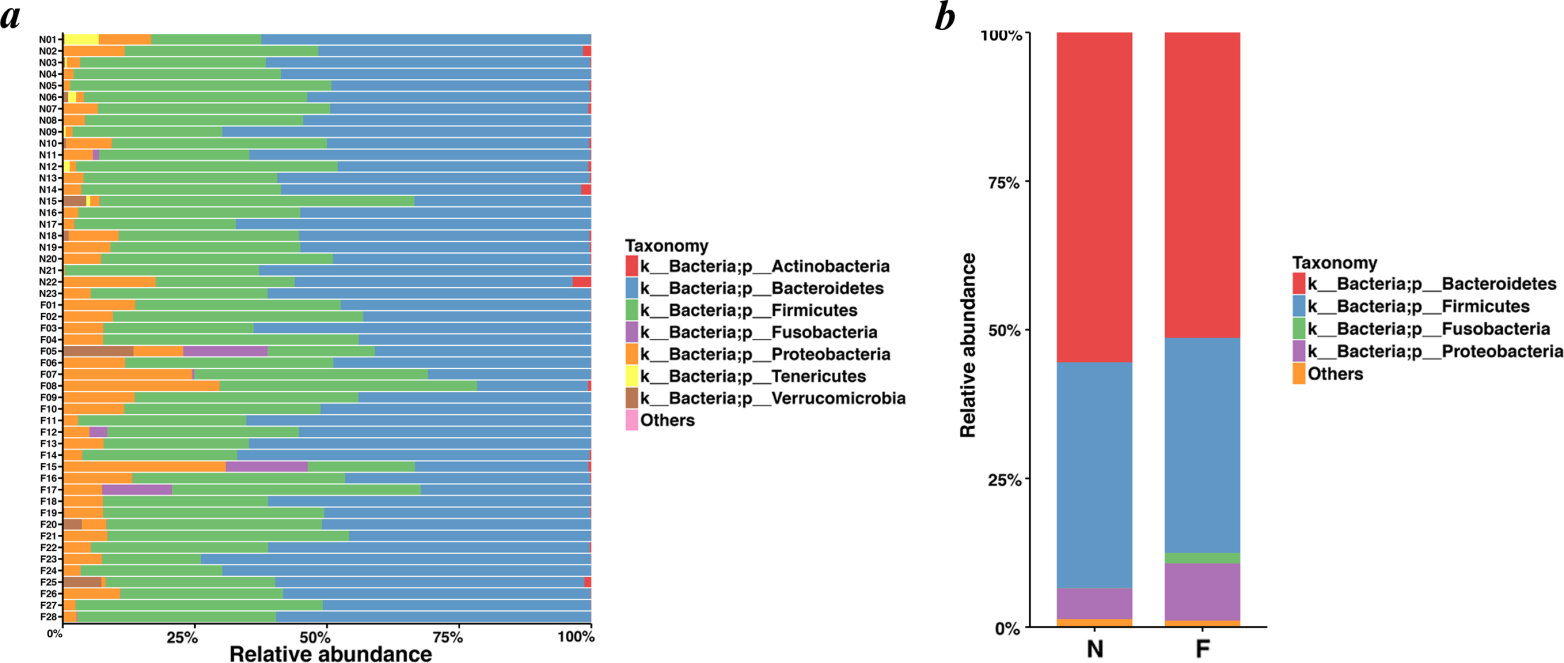
Histogram of the community composition of gut microbiota at the phylum level. (A) The ordinate represents the sample, and the abscissa represents the relative abundance. (B) The abscissa represents the group, and the ordinate represents the relative abundance. The figures show species with a relative abundance of 1% or more. All species with a relative abundance of less than 1% and classified as “unclassified” and “unidentified” were classified as “Others”. (N: normal weight group; F: obese group).

Bacteroidetes was the most predominant phylum, contributing 55.48% and 51.35% of the gut microbiota in the normal weight group and the obese group, respectively, followed by Firmicutes, contributing 37.93% and 36.18%, respectively (Figs. 4A and 4B). Proteobacteria, Fusobacteria, Verrucomicrobia and Actinobacteria constituted the next most dominant phyla. Microbial compositions showed high interindividual variability, among which Bacteroidetes accounted for 21.01-73.78%, and Firmicutes accounted for 18.72-59.49% among all individuals. In regard to phyla, proportion of Bacteroidetes (51.35%) in the obese group was lower than that in the normal weight group (55.48%) (*P* = 0.030, Wilcoxon rank-sum test). No statistical differences were revealed in Firmicutes (*P* = 0.436, Wilcoxon rank-sum test) and the Firmicutes/Bacteroidetes (*P* = 0.983, Wilcoxon rank-sum test) between the two groups.

Analysis of the relative abundance of bacterial taxonomic groups showed that the fecal microbiota was dominated by fourteen genera: *Bacteroides* (mean relative abundance, N, 41.58%; F, 40.06%) (In the following analysis, ”N” is short for “Normal weight” and “F” for “Fat”, namely “Obesity”), *Faecalibacterium* (N, 7.03%; F, 10.76%), *Prevotella* (N, 4.72%; F, 6.19%), *Oscillospira* (N, 4.50%; F, 1.61%), *Dialister* (N, 4.03%; F, 0.91%), *Parabacteroides* (N, 4.01%; F, 2.39%), *Sutterella* (N, 2.68%; F, 5.60%), *Roseburia* (N, 1.94%; F, 2.32%), *Ruminococcus* (N, 1.91%; F, 1.13%), *Phascolarctobacterium* (N, 1.64%; F, 3.43%), *Lachnospira* (N, 1.55%; F, 3.23%), *Escherichia* (N, 1.39%; F, 1.32%), *Megamonas* (N, 0.54%; F, 1.79%), *Haemophilus* (N, 0.32%; F, 1.96%). *Faecalibacterium, Phascolarctobacterium, Lachnospira*, *Megamonas*, and *Haemophilus* in the obese group were significantly more abundant than those in the normal weight group (*P* = 0.048, *P* = 0.018, *P* <0.001, *P* = 0.040 and *P* = 0.003, respectively, Wilcoxon rank-sum test). As for *Oscillospira* and *Dialister*, they took lower proportions in the obese group when compared to the normal weight group (*P* = 0.002, two-tailed *t*-test; and *P* = 0.002, Wilcoxon rank-sum test). For the remaining genera, the gut microbiome sequencing data did not indicate significant differences in the abundance for the obese group compared to the normal weight group.

### Phylogenetic and taxonomic profiles of gut microbiota and PICRUSt analysis

**Figure 5:**
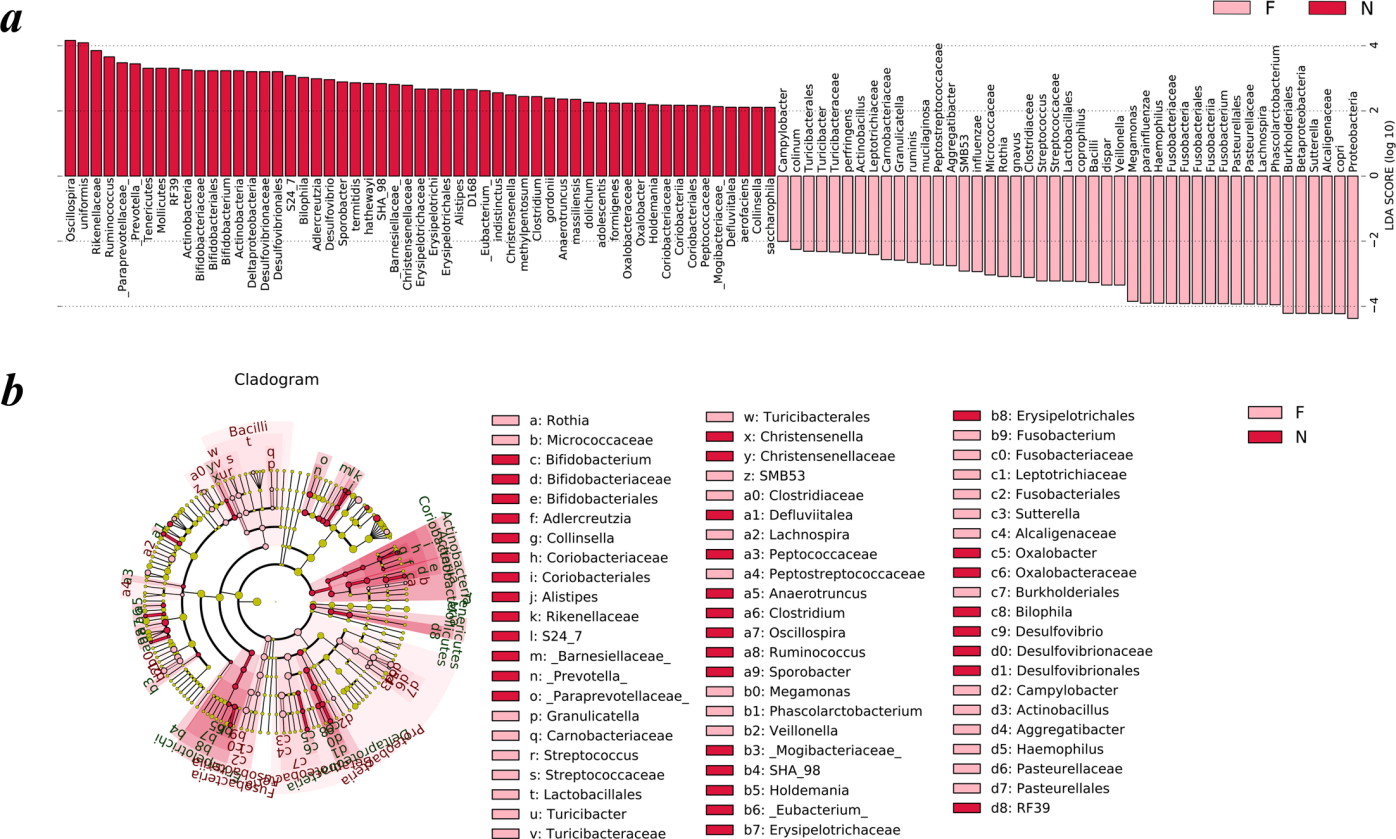
Different structures of gut microbiota in the two groups. The bar graph and cladogram indicate the taxa that discriminate among the two groups , based on the LEfSe method and linear discriminant analysis (LDA) effect size method. (A) The statistical test was performed using the LDA effect size method. Only taxa with an alpha value of 0.05 and with absolute LDA (log10) scores >2.0 were considered significant. (B) Cladogram depicting the phylogenetic distribution of microbial lineages associated with the two groups. Each small circle at a different classification level represents a classification at that level, and the diameter of the small circle is proportional to the relative abundance (the levels represent, from the inner to outer rings, genus, family, order, class and phylum). (N: normal weight group; F: obese group).

To analyze the statistical differences in microbial communities between the normal weight group and the obese group, we compared OTUs with the LEfSe analysis. The histogram reflected the LDA scores that were computed for the features at the OTU level (Fig. 5A). Cladograms of the taxa with LDA values >2.0 were depicted in Fig. 5B.

Upon analyzing the fecal samples, a total of 55 species of bacteria were abundant in the normal weight group and 45 species were abundant in the obese group. As shown in Fig. 5A, the relative abundance of taxonomic groups showing an LDA score greater than 10^4^ was summed for the normal weight group (*Oscillospira* and *Bacteroides uniformis*) and the obese group (Proteobacteria, *Prevotella copri*, Alcaligenaceae, *Sutterella*, Betaproteobacteria, and Burkholderiales). Multiple genera were found in significantly high abundances in the normal weight group. These included *Oscillospira*, *Ruminococcus*, *Prevotella*, *Adlercreutzia*, *Sporobacter*, *Bifidobacterium*, *Clostridium*, *Desulfovibrio*, *Bilophila*, *Christensenella*, *Alistipes*, *Anaerotruncus*, *Eubacterium*, *Holdemania*, *Oxalobacter*, *Defluviitalea*, and *Collinsella*. The genera that were enriched in the obese group were *Turicibacter*, *Campylobacter*, *Actinobacillus*, *Aggregatibacter*, *SMB53*, *Rothia*, *Granulicatella*, *Streptococcus*, *Veillonella*, *Megamonas*, *Fusobacterium*, *Phascolarctobacterium*, *Haemophilus*, *Lachnospira*, and *Sutterella*.

Cladograms (Fig. 5B) were generated from the LEfSe analysis, which showed the most differentially abundant taxa enriched in microbiota with green for the normal weight group and red for the obese group. The diameter of each circle is proportional to its abundance. The obese group showed a significant decrease in the phylum Actinobacteria, phylum Tenericutes, class Deltaproteobacteria, class Erysipelotrichia and some taxonomic groups belonging to the order Bacteroidales, such as family Paraprevotellaceae, Barnesiellaceae, S24-7, and Rikenellaceae and a greater abundance of the phylum Proteobacteria, phylum Fusobacteria and class Bacilli when compared with the normal weight group (Fig. 5B).

To study the changes of gut microbial function in obese children, we adopted PICRUSt. Based on KEGG database, PICRUSt revealed a total of six biological metabolism pathways at Level 1 pathways: metabolism, genetic information processing, environmental information processing, cellular processes, organismal systems, human diseases. Among them, metabolism, genetic information processing and environmental information processing dominated, accounting for 46.89%-51.41%, 18.33%-22.12% and 10.22%-14.87%, respectively. These six pathways in the obese group were lower than those in the normal weight group. Meanwhile, the secondary function of the predicted gene was analyzed, finding it consisted of 39 sub-functions including membrane transport, carbohydrate metabolism, amino acid metabolism, replication and repair, energy metabolism, translation, cellular processes and signaling. Within the 39 predicted functional categories at KEGG pathway hierarchy level 2, except immune system diseases, neurodegenerative diseases and signal transduction, the remaining 36 sub-functions within the predicted gene all decreased in obese children.

## Discussion

Gut microbiota plays is essential to regulate energy metabolism and fat storage and is closely associated with the occurrence and development of obesity (MetaHIT Consortium, 2011; Collins et al., 2015; Musso, Gambino & Cassader, 2010; Walters, Xu & Knight, 2014). Studies have confirmed that the composition of gut microbiota changes and the microbial diversity decreases in obese people and obese rats (Petriz et al., 2014). In the present study we found alterations in gut microbiota composition in the obese and normal-weight Chinese children. Rarefaction curves and rank-abundance curve suggested the abundance and diversity of gut microbiota in obese children were lower than those in normal weight children and. Alpha diversity metrics generated a more accurate verification. Alpha diversity is an index reflecting the variety of microbial species in stool samples. A higher alpha diversity indicates higher abundance in one sample (Liu et al., 2017). In this study, the alpha diversity indices, including Chao1, observed species, PD whole tree and Shannon index, revealed that the gut microbial composition of children in different BMI categories was statistically significant. The abundance and diversity of gut microbiota in obese children were significantly lower than those in normal weight children, which is consistent with existing research (Le Chatelier et al., 2013; Gao et al., 2017; Menni et al., 2017; Scheithauer et al., 2016). Such differences are attributed to the excessive intake of high fat foods by obese children (Tilg & Moschen, 2014). A high-fat diet can reduce the expression of the intestinal epithelial tight junction proteins occludin and ZO-1, affect the integrity of the intestinal epithelium, cause changes in intestinal permeability and increase the level of circulating lipopolysaccharide, which contribute to increase the incidence of obesity (Cani et al., 2008). Beta diversity is an index reflecting the heterogeneity of gut microbiota between samples in each group. A higher beta diversity is indicative of larger compositional differences in the gut microbiota between samples in a certain group (Liu et al., 2017). We used unweighted UniFrac analysis to compare the similarity and heterogeneity among gut microbial communities and identified an apparent clustering pattern in the normal weight group and the obese group. The normal weight group and the obese group were distinct from each other in terms of gut microbiota composition, it showed that the structure of gut microbiota changed significantly with body weight, and these changes may be associated with the occurrence and development of obesity.

In the gut of healthy humans, intestinal microorganisms can be divided into six phyla: Firmicutes, Bacteroidetes, Proteobacteria, Actinobacteria, Fusobacteria and Verrucomicrobia (Eckburg et al., 2005; Lozupone et al., 2012), among which Firmicutes and Bacteroidetes accounted for more than 90% (Mariat et al., 2009). We also found that the top 6 phyla of gut microbiota in children were Bacteroidetes, Firmicutes, Proteobacteria, Fusobacteria, Verrucomicrobia and Actinobacteria, among which Firmicutes and Bacteroidetes accounted for approximately 90%. Research regarding the changes in Bacteroidetes and Firmicutes in gut microbiota is not settled. Some studies found that Bacteroidetes in obese children decreased while Firmicutes increased (Rahat-Rozenbloom et al., 2014; Turnbaugh et al., 2008; Patrone et al., 2016), while others revealed that the number of both Bacteroidetes and Firmicutes in obese children increased (Ismail et al., 2011). This study found that compared with the normal weight group, the number of Bacteroidetes in the obese group was significantly reduced, which was consistent with the results of Ley et al. (Krajmalnik-Brown et al., 2012; Ley et al., 2006; Turnbaugh et al., 2008). In the healthy gut, Bacteroidetes plays an essential role in degraded plant polysaccharides that cannot be absorbed in the human body and participating in the nutrition metabolism of the human body together with other bacteria. A long-term high-fat diet will reduce the number of Bacteroidetes, affect the absorption of polysaccharides and proteins, and hence result in obesity (Murphy et al., 2010). In contrast, this study found that the number of Firmicutes did not change significantly. Finucane et al. also found no difference between obese versus lean individuals in their relative abundance of Firmicutes (Finucane et al., 2014). This can be explained by the differences in ethnic groups, diet and lifestyle of the subjects (De Filippo et al., 2010; Zhang et al., 2013; Matsuyama et al., 2019; Wu et al., 2011; Bai, Hu & Briner, 2019). Additionally, the ratio of Firmicutes to Bacteroidetes (F/B) is often regarded as a marker of obesity in related studies; especially the F/B value in obese animals is higher than normal weight, but this result does not apply to the study of the human body (Finucane et al., 2014; Ley et al., 2005). This study found that the F/B value was significantly different among different individuals, indicating that not all obese individuals had significantly increased F/B values, and the F/B ratio between the two groups had no statistical difference, which confirmed other relevant reports (Karlsson et al., 2012; Schwiertz et al., 2010). However, studies on the gut microbiota of obese children in the Antwerp region of Belgium and the Kazak region of Xinjiang found that the F/B ratio increased (Bervoets et al., 2013; Xu et al., 2012). To sum up, currently, there are ambiguities in the relationship between the changes in the F/B ratio and the incidence of obesity. Research conclusions on this aspect are not consistent all over the world, mainly because the effect of gut microbiota on obesity is far more complicated than the imbalance or interaction of two microbial phyla. Therefore, whether it is appropriate to simply use the F/B ratio as a marker of obesity remains to be discussed. More detailed research is needed to evaluate the relationship between the F/B ratio and obesity.

To investigate the relationship between gut microbiome functions and obesity, we predicted the potential metagenomes with PICRUSt. The inferred gene families were annotated and combined with level 1 to level 3 pathways. After analyzing the predicted gene copy number of gut microbiota in different groups, we found that the relative abundance of gut microbiota in obese children changed, and their functions also changed correspondingly. The overall trend of predicted gene copy number in the Level 1to Level 3 was lower than that in the normal weight group. Unfortunately, these changes were not statistically significant. A larger sample size will help to carry out in-depth research on this aspect.

To further study the difference in gut microbiota in children with different body weights, LEfSe analysis based on the OUT level was conducted to screen key biomarker species. At the genus level, significant differences existed in the composition of some microorganisms between the normal weight group and the obese group. LDA difference analysis of intestinal microflora in the two groups was conducted to compare strains with LDA scores greater than 2. The results showed that the genera *Oscillospira*, *Ruminococcus*, *Prevotella*, *Adlercreutzia*, *Sporobacter*, *Bifidobacterium*, *Clostridium*, *Desulfovibrio*, *Bilophila*, *Christensenella*, *Alistipes*, *Anaerotruncus*, *Eubacterium*, *Holdemania*, *Oxalobacter*, *Defluviitalea*, and *Collinsella* were significantly higher in the gut microbiome of the normal weight group. The genera *Turicibacter*, *Campylobacter*, *Actinobacillus*, *Aggregatibacter*, *SMB53*, *Rothia*, *Granulicatella*, *Streptococcus*, *Veillonella*, *Megamonas*, *Fusobacterium*, *Phascolarctobacterium*, *Haemophilus*, *Lachnospira*, and *Sutterella* were enriched in the obese group. A recent study from Canada discovered that the abundance of *Oscillospira*, which is closely related to the decline of childhood obesity, increased in the infant’s gut microbiota three months after birth or when the expectant mother was in contact with pet animals (Tun et al., 2017). Sclerenchyma contains a large number of bacteria with fermentation functions, such as *Ruminococcus*, which can decompose food fibers that cannot be digested by the human body into absorbable short-chain fatty acids (acetic acid, propionic acid, butyric acid, lactic acid, etc.) and increase energy intake through intestinal absorption (Turnbaugh et al., 2006). Moreover, the short-chain fatty acids produced by fermentation can also act on the G protein-coupled receptors 41 and 43 to promote intestinal endocrine cells (L cells and I cells) to synthesize and secrete peptide YY, PYY and glucagon-like peptide-1 (GLP-1), slow intestinal peristalsis and promote sugar-induced insulin secretion, eventually leading to energy concentration and fat accumulation (Duca, Sakar & Covasa, 2013). Cani et al. (2007) showed that a high-fat diet led to a decrease in the number of *Eubacterium*, *Clostridium* and *Bifidobacterium* and simultaneously caused inflammation, obesity and insulin resistance. Hao et al. analyzed the bacterial content in feces of obese people in China. The results showed that compared with normal weight people, the number of *Bifidobacterium* in obese people had a decreasing trend (Zuo et al., 2011). Recent studies have reached the same conclusion (Gao et al., 2017). Compared with the control group, Wang et al. found that in a high-fat diet group, the weights of SD rats increased with the numbers of *Lactobacillus* and *Bifidobacterium* in the intestinal tract dropped significantly, while the numbers of *Bacteroides* and *Clostridium* showed an upward trend, especially *Clostridium* (Wang et al., 2013; Bao et al., 2012). *Clostridium* can upregulate the expression of glucose transporter 2 in the jejunal mucosa and the expression of lipoyltransferase in the ileal mucosa, which can lead to an increase in the absorption of glucose and fats (Woting et al., 2014). Some studies also showed that *Clostridium* was negatively correlated with fasting blood glucose, HbA1c and insulin levels (Karlsson et al., 2013b). Gao et al. documented that the abundance of *Fusobacterium* was significantly higher in obese people (Gao et al., 2017). A study in 2014 showed that compared with plant foods (e.g., fruits and vegetables), high-fat animal foods (e.g., meat, eggs and milk) had a greater impact on gut microbiota. In the intestinal tracts of people that eat animal foods, bacteria that were resistant to cholate, such as *Bilophila*, grew significantly (David et al., 2014). *Sutterella* was significantly high in the obese group in a study of the relationship of gut microbiota and diarrhea, autism and eczema (Finegold, 2011; Lv et al., 2017). Some studies have noted that *Streptococcus* is related to Crohn’s disease (CD), and *Streptococcus* is of great importance in the inflammatory mucosal region of CD patients (Fyderek et al., 2009). *Veillonella* was able to decompose glucose and lactic acid into short-chain fatty acids (Burger-van Paassen et al., 2009). However, these short-chain fatty acids cannot synthesize mucoprotein but can lead to the increased permeability of intestinal mucosa and facilitate the formation of inflammation (Brown et al., 2011). Some researchers indicted that the species of *Lachnospira* might be associated with type 2 diabetes (Kameyama & Itoii, 2014). However, there are few reports on the relationship between *Bilophila*, *Streptococcus*, *Veillonella*, *Lachnospira* and obesity, which warrants further research. In brief, our findings provide further support for the fact that the increase and decrease of these key biomarker species are closely associated with obesity. Our findings may also suggest potentially hazardous microorganisms. In the future, studies carried on larger sample size will be conducive to illustrate the function of gut microbiome composition in influencing the BMI.

Several limitations in our study should be considered. It was conducted in cross-section and could not provide evidence of a causal effect between the gut microbiota and obesity. With the small sample sizes, we cannot rule out the possibility of chances in our findings. Thus, validation on a larger sample size is needed. High-throughput sequencing technology requires high quality samples and the DNA concentration of whole genome in some sample bacteria cannot reach amplification requirements. Subjects selected for this study only represent the gut microbial composition of children in one specific region. In spite of the inclusion and exclusion criteria, the results were still influenced by the factors of subjects themselves, the individual differences among the subjects were apparent. Gut microbial samples in this study were all from one certain point in time, but it is currently considered that a long-term observation may be more valuable to study the dynamically changing gut microbiota.

## Conclusions

The gut microbiota composition of obese children is quite different from that of normal weight people, and intestinal dysbiosis has a close relationship with the occurrence and development of obesity. However, there are still many inconsistencies at present, which requires a large number of studies to verify the causality between specific intestinal bacterial species and obesity, and the relevant mechanism needs to be explored more deeply and accurately. On the other hand, as far as existing studies are concerned, an overwhelming majority of experimental results are obtained from animal experiments, but there are few studies on the mechanism and flora in the human body. Both gut microbiota and obesity are affected by a variety of factors. It will be of far-reaching significance to expound on their acting mechanism and incorporate multiple variables into long-term clinical studies in a reasonable way. Upon diagnosis, gut microbiota is a therapeutic target of obesity. The regulation of the composition of gut microbiota or the regulation of the production of gut microbiota metabolites, including the transplant of host bacteria, the use of antibiotics, biological bacterial agents, food and drug treatment, will bring new ideas to obesity treatment, but we still need a large number of medical studies to support them.

##  Supplemental Information

10.7717/peerj.8317/supp-1 Supplemental Information 1 Rarefaction curves and rank abundance for calculated OTUs

10.7717/peerj.8317/supp-2 Supplemental Information 2 Detailed information of 16S rRNAgene sequences for all samples

10.7717/peerj.8317/supp-3 Supplemental Information 3 Relative abundances of gut microbiota at the phylum level

10.7717/peerj.8317/supp-4 Supplemental Information 4 Relative abundances of gut microbiota at the genera level

10.7717/peerj.8317/supp-5 Supplemental Information 5 Chao1 indices of bacterial communities

10.7717/peerj.8317/supp-6 Supplemental Information 6 Observed species of fecal bacterial communities

10.7717/peerj.8317/supp-7 Supplemental Information 7 PD whole tree of fecal bacterial communities

10.7717/peerj.8317/supp-8 Supplemental Information 8 Shannon index of fecal bacterial communities

10.7717/peerj.8317/supp-9 Supplemental Information 9 Detailed information of LEfSe

10.7717/peerj.8317/supp-10 Supplemental Information 10 Level 1 pathway

10.7717/peerj.8317/supp-11 Supplemental Information 11 Level 2 pathway

10.7717/peerj.8317/supp-12 Supplemental Information 12 Level 3 pathway

## Additional Information and Declarations

### Data Availability

The following information was supplied regarding data availability: The raw data is available in the Supplementary Files.
