# Supplementary figures and images for: Alteration of the gut microbiota associated with childhood obesity by 16S rRNA gene sequencing
Source: PeerJ. 2020 Jan 14;8:e8317. doi: 10.7717/peerj.8317 (PMC6968493; doi:10.7717/peerj.8317)

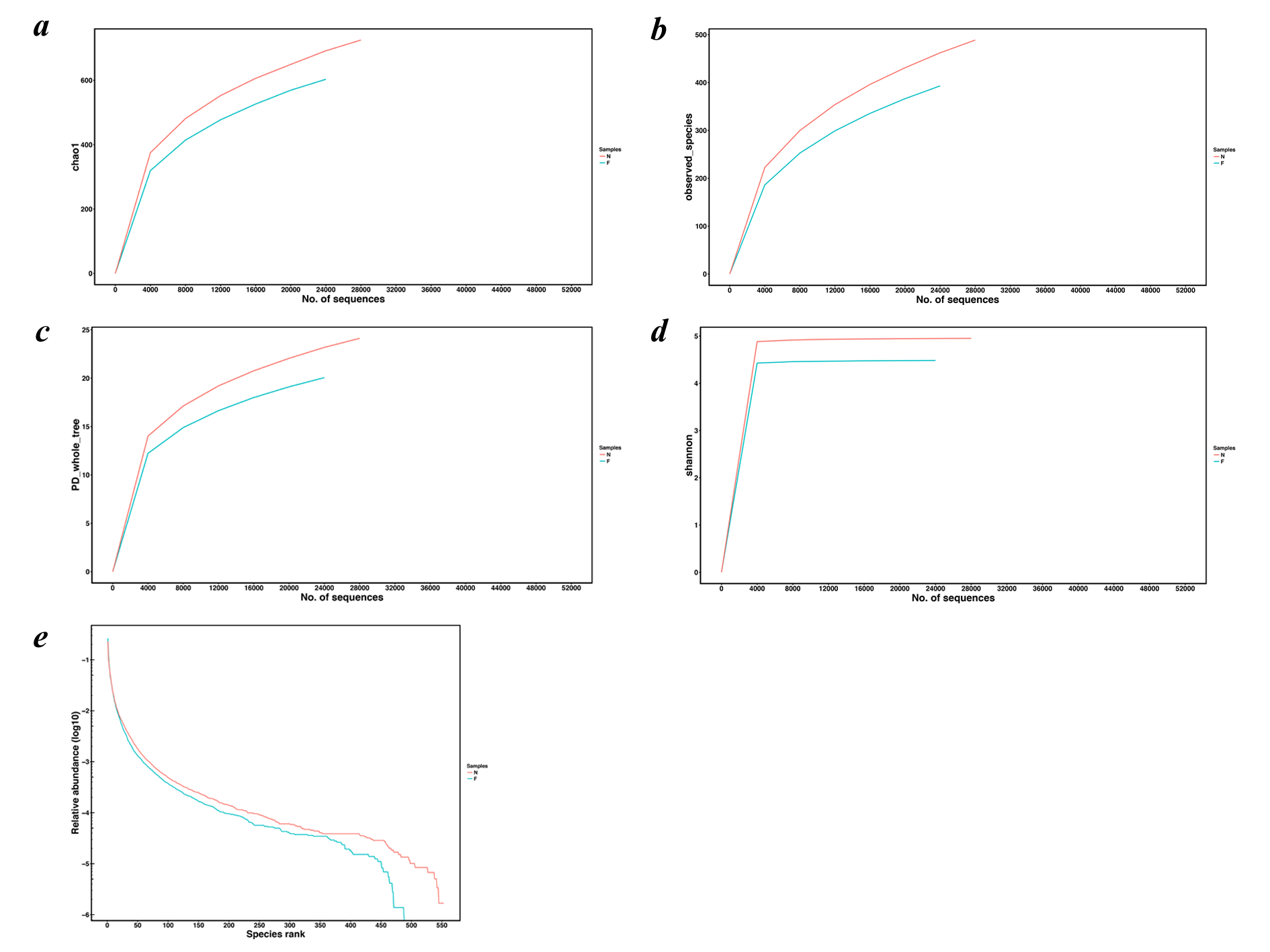

Supplement: Supplemental Information 1 [file peerj-08-8317-s001.png]
